# School Bullying in Compulsory and Advanced Secondary Education. Determining Factors in its Intervention

**DOI:** 10.3390/ijerph16050750

**Published:** 2019-03-01

**Authors:** Isabel Martínez Sánchez, Rosa Goig Martínez, Daniel González González, José Álvarez Rodríguez

**Affiliations:** 1 Department of Methods of Research and Diagnosis in Education I (MIDE I), Faculty of Education, Spanish University for Distance Education (UNED), 28040 Madrid, Spain; rmgoig@edu.uned.es; 2 Department of Methods of Research and Diagnosis in Education (MIDE), Faculty of Education, Universidad de Granada, 18071 Granada, Spain; danielg@ugr.es; 3 Department of Pedagogy, Faculty of Education, Universidad de Granada, 18071 Granada, Spain; alvarez@ugr.es

**Keywords:** school bullying, intervention, school climate, prevention, coexistence, peer relations

## Abstract

*Background*: School bullying is a problem that has been considered from various different perspectives in the academic literature. The present work seeks to increase knowledge on the influence of the climate established at the school in order to determine if contextual factors can be used as a reference from which to plan interventions directed towards prevention. *Methods*: An ad hoc validated questionnaire was administered to 554 pupils in secondary education (compulsory and A Level) who were aged between 12 and 18 years. *Results*: Descriptive bivariate analysis showed the quality of the relationship established by the individual with their environment to be a key indicator of their susceptibility to school bullying. In the same way, acceptance in school is a protective factor against victimization. *Conclusions*: The most effective interventions are those which focus on the center of education and involve all of the educational community, taking a transversal approach that transforms the cultural system within which students develop.

## 1. Introduction

School bullying is an important social problem [1,2,3] and presents a threat to the wellbeing of all members of the school or educational center [3,4]. In recent years awareness and visibility of bullying have increased in schools and in the media [5,6,7].

The World Health Organization (WHO) has called for further research into bullying and has stressed the seriousness of this phenomenon. It considers it to be a first-order problem due to the seriousness of consequences for all individuals who are affected by it in one way or another [3,8]. A number of authors, including Hymel and Swearer (2015), Saarento, Garandeau, and Salmivalli (2015) and Hall and Chapman (2018) [2,4,9] amongst others, agree that the social nature of this problem means that there are no differences in prevalence based on the type of school environment or the socioeconomic context. It is instead recognized as a general problem that exists in all types of backgrounds.

As has been discussed by López et al. (2018) [10], school bullying in Spain is a priority for the educational system, which has to channel its efforts towards improving the climate within the school with a view to reducing conflict. At the same time, the educational system must consider more traditional problems such as those related with student academic performance and the quality of the education delivered. School bullying has been comprehensively defined, documented, and analyzed from various academic standpoints, having been described as a serious social problem that is characterized by direct (such as physical intimidation and verbal threats) and indirect (exclusion, rejection) [8,11] aggressive behaviors.

## 2. Theoretical Framework

Hong and Espelage (2012) [1] have highlighted that the definitions of school bullying employed in research studies differ as a function of the perspective taken in each research study, with certain aspects having been noted as being especially inconstant. Hall and Chapman (2018) [4] corroborate this viewpoint referring to existing differences in the conceptualization of bullying across countries, with these differences being identified in both theoretical and empirical research studies. Further, pioneer of the field Olweus, in his first definition of the phenomenon focused on identifying who the bully was and how they behaved. Other studies that followed on from this, such as that conducted by Griffin and Gross in 2004, sought to characterize the relational dynamics in which these behaviors occur [1]. According to Hall and Chapman (2018) [4], school bullying is defined by three characteristics that are found in all studies developed to examine this phenomenon and correspond to contemporary perspectives of it: 1. The intention of the aggressor to cause harm to the victim; 2. the imbalance of power, created due to the aggressor being physically stronger and/or having more social power than the victim, and 3. repetition of the acts of bullying.

In the case of bullying, acts of intimidation take place when an individual or a group of individuals, in exercising a dominant role in relation to another person who is deemed to be imponent, cause repeated and intentional harm to that individual. These acts can vary in typology and it must be noted that not all conducts are violent per se, though they do share common results with regards to outcomes for the victim [12]. Consequently, the definition of school bullying excludes those phenomena that do not include all of the aforementioned characteristics, such as isolated fights arising from conflicts of interests. Hall and Chapman (2018) [4] argue that it therefore follows that the definition of school bullying should incorporate undesirable and aggressive behaviors encountered by students and enabled through the use of power directed to cause physical and/or psychological harm. These typically occur in the victim’s environment, which is usually the school.

School bullying is present in all contexts as a social dynamic [1,11,13,14] and has reached a large prevalence in secondary education (compulsory secondary education in Spain consists of courses from first to fourth grade (from 12 to 16 years old); in Australia, secondary school runs from Year 8 to Year 10 (from 13 to 16 years old) and is compulsory up until Year 9 (until 15 years old). In the United States and Canada, these courses are equivalent to all grades from grade 7 up until grade 10.) and baccalaureate (The baccalaureate (or higher secondary education) in Spain is formed by first and second grade courses, being equivalent to grades 11 and 12 in the United States and Canada. In Australia, these studies correspond to those undertaken in Year 11 and Year 12 of secondary school (from 16 to 18 years old).) education. It has been documented that at least 22% of students in the United States have found themselves in one of the aforementioned associated roles (aggressor or victim) at some moment during compulsory school education [11,15]. In the European context, prevalence has been reported at between 20% and 30% [14]. According to Nacimiento and Mora (2014) [16], around 6% of students in secondary and higher education in Spain are involved in this phenomenon and 83% report having witnessed, on some occasion, some circumstance that could be considered to be an integral part of bullying, using the definition offered. According to García, Pérez, and Nevot (2010) [17] these data highlight the magnitude of this phenomenon and the need to investigate the mechanisms that could prove to be efficacious for reducing the prevalence and severity of consequences. According to Hall and Chapman (2018) [4], consequences are suffered by every individual who comes into contact with this reality, although the most serious consequences are suffered by the victimized subjects. Victims of school bullying often feel insecure and unhappy at school. This can result in disconnection from the school, absenteeism, and deteriorations in academic performance due to problems in concentration and attention [4]. Further, the consequences of school bullying can also reach a tipping point that causes mental health problems in victims. These can be experienced in both the short and long term [4].

With regards to the seriousness of these effects, the need to deliver active prevention in connection with targeted interventions that tackle the episodes presented, has been highlighted to encourage reductions in these behaviors [18]. When designing these interventions, it is essential to identify the characteristics of the individuals involved as much as it is to identify the environment [19], an aspect upon which the present study was developed in the context of secondary and baccalaureate education. Norwalk et al. (2016) [20], similar to the present study, focused on the period of adolescence and found it to be a stage of vulnerability characterized by physical, psychological, and social changes. According to Norwalk et al. (2016) [20], these changes must be held in mind given the consequences that they will have on the school climate. Likewise, findings reported by Norwalk et al. (2016) [20] indicate that given the fact that young people spend more time at school or in school-related activities, schools and educational centers become important contexts for socioeconomic, academic, and behavioral adjustment. This makes it imperative that schools act to create a context capable of minimizing acts of bullying, in addition to laying cultural and political foundations in the institution which align with the prevention of school bullying.

Following Hong and Espelage (2012) [1], an efficacious preventative strategy should take an ecological approach. This should also consider the way in which all the individual variables that influence the context in which they are applied, are programmed. In this regard, a number of studies such as those conducted by Martínez-Otero (2005), Del Rey, Ortega, and Feria (2009), and Álvarez-García, García, and Núñez (2015) [21,22,23], have highlighted the importance of analyzing coexistence in schools as a way of detecting potential risk factors that arise during conflicts. This problem is examined in the present study. Espelage (2014) [24] employs the ecological systems theory of Bronfenbrenner as a framework in their research to analyze the interaction between risk factors and protective factors during infancy and adolescence, further individualizing the roles of family, the school, and society in school bullying. The interaction between the components of a microsystem is known as a mesosystem. This provides information on how context can act to exacerbate or cushion experiences of the young people who participate in intimidation (for example, family support can mitigate the impact of victimization amongst peers). This led Espelage (2014) [24] to establish some key considerations when planning for prevention, such as the emotional needs of pupils. 

Hall and Chapman (2018) [4] have also highlighted the relevance of socioeconomic factors linked to the school climate when designing prevention strategies. They outline the importance of establishing quality relationships between the individuals that make up the educational community. The relationship formed between the teachers and their pupils, specifically, is key given the responsibility both have to construct a pleasant and respectful atmosphere at the school. According to Hall and Chapman (2018) [4] it is also of interest that the teachers are conscious of the resources available to them for improving the school climate and, as a result, improving coexistence in the school.

The concept of coexistence has been shown to correlate with the intrinsic elements of any given social relationship, such as respect and tolerance, which must be present in order to generate a positive climate [22]. Nevertheless, in practice, it is obvious that coexistence in schools can present difficulties that alter the climate and are perceived by the individuals who are developing within the environment. Depending on the resulting perceptions, possible repercussions are conflict and the emergence of acts of school bullying. As reported by Del Rey, Ortega, and Feria (2009) [22], the degree of school conflict is an indicator on which proposed interventions can be grounded. In the same sense, Valdés and Martínez (2014) [25] argue that such interventions should be designed using an ecological perspective in which all aspects that make up the complexity of relationships are taken into account.

Further, it will also be necessary to consider the individual consequences of school bullying for both the victims and the other implicated individuals. In this regard, Menesini, Modena, and Tani (2009) [26] have reported findings regarding the set of symptoms for both the victims and the aggressors, which compromise the emotional wellbeing of both, in both the short term and into adulthood. According to Joffre et al. (2011) [27] this has further implications on the quality of life of all who experience it. For this reason, it is crucial to advance prevention approaches in order to eradicate the associated negative implications. In light of the previously discussed research, the present study seeks to understand the profile of a bully in secondary schools. Further, it analyzes consequences for the victims and the other implicated individuals, and seeks to identify the motives and causes behind the increase seen in recent years in school bullying amongst peers in the examined locality. In connection with these goals, the most common profiles of bullies in secondary school classrooms will be presented and the types of interventions carried out to detect, prevent, or eradicate school bullying will be described. Similarly, the motives of the victims and other implicated individuals for not reporting occurrences of bullying will be uncovered and the profiles of each class according to the situations reported in their school will be documented. The hypotheses of the study are as follows:

**Hypothesis** **1** **(H1).** *Contextual factors influence school bullying, interventions designed to prevent this problem should be informed by these variables.*

**Hypothesis** **2** **(H2).** *Contextual factors do not influence the phenomenon of school bullying, it will follow that the proposed interventions can be applied in any scenario and enable generalization.*

## 3. Materials and Methods

The present study employs a quantitative and descriptive design which enables examination of a wide group of people through inferential statistics to produce by independence testing. This permits analysis of all of the proposed variables in both an isolated and interactive way [28]. To achieve this, an instrument was designed through which opinions regarding key aspects of the problem originating from diversity and the relationship of these aspects with school bullying, could be more closely examined.

### 3.1. Participants

The sample used for data collection was formed of 554 pupils aged between 12 and 18 years, who were undertaking compulsory secondary or baccalaureate education at the same educational center, at the time of data collection. For recruitment, convenience sampling was used that enabled accessible cases to be included who consented to be included in the study.

The distribution of data reported by participants is presented in Table 1.

The sample is formed of 51.6% females and 48.4% males. With regards to the type of course being attended, the largest group included in the present study pertained to the first year of baccalaureate study, making up 22.6% of the participants in the present study (Table 2). Chi-Squared analysis of the variables sex and course signaled the presence of an association between both dimensions. Females represented a higher proportion of pupils enrolled on all of the various courses, apart from on the third grade of CSE. In this case, the number of males was almost twice as many as females (4.9% of all study participants were females on this course, relative to 9.2% being males on this course).

With respect to the family nucleus, 84.7% indicated that they were living with their father and mother, with only 10% of participants reporting to live with only one of their parents. A total of 61.6% reported having one sibling, 18.8% reported having two siblings, and 6.7% had three or more. The variables describing the number of siblings was shown to be independent with respect to the variable describing sex, with no association being found between them.

### 3.2. Materials

A questionnaire was designed through which pupil opinion regarding key aspects of problems originating from diversity and their relationship with school bullying could be better understood. Being a non-invasive questionnaire, only verbal consent was secured. The instrument, which was developed specifically for the present study, was created in response to calls to develop a specific tool capable of carrying out an approach of this nature, given that no other appropriate questionnaires could be identified.

For the development of the questionnaire the following steps were taken:

1. Literature review.
2. Study of dimensions and selection of indicators.
3. Development of a battery of items corresponding to each dimension.
4. Development of the first version of the questionnaire.
5. Evaluation of the questionnaire: Expert panel.
6. Development of the definitive questionnaire incorporating the suggestions received.

Once the literature review of the topic was conducted, we developed the instrument using the questionnaire for students and teachers on the initial state of school coexistence by Ortega and Del Rey (2003) and the questionnaire of intimidation and abuse amongst peers in secondary school by Ortega, Mora-Merchán, and Mora (2005), as starting points. We observed that these questionnaires contained relevant items which allowed us to obtain the necessary information to develop the present study. Whilst these questionnaires have been validated by the scientific community, we embarked upon a process of validation given that the aim of the study was to develop a new questionnaire specific for the present research.

To this end, once the first version of the instrument was developed, its validation by experts on the material was initiated. In instances when the evaluation of items by the various experts differed, the aim was to reach an agreement on the nature of required changes (Pérez Juste y García Ramos, 1989) [29]. The distributed questionnaire was divided between 2 dimensions: (1) Evaluation of the items (example: —the phrasing of the item is formulated correctly, —the phrasing of the item is understandable for participants and (2) evaluation of the response options (example: the alternatives for the response provide sufficient information relating to the question, —the different response options are coherent with the phrasing).

Specifically, the developed tool was validated by 9 experts composed of lecturers from the Faculty of Education Science who provided expertise in the field of methodology and teachers of secondary and baccalaureate education qualified in therapeutic pedagogy, who provided expertise in the field of attention to diversity. The score obtained for this questionnaire using the method described exceeded the cut-off point of 27, which had been established for supporting the adequacy of the instrument (each expert assessed on a scale of 1 to 4, with 1 being no fit of the proposed tool and 4 being a high degree of fit).

Given that the included variables relate to opinions reported by participants, this type of content validity is sufficient to support the use of the questionnaire as a data collection instrument in the present study. However, factor analysis was conducted to confirm the default structure of the questionnaire. Reliability of presented inferences was examined through estimations of global internal consistency of all items of the instrument. This was achieved by calculating the Cronbach alpha coefficient using the statistical software program, SPSS (IBM, Armonk, NY, USA). This obtained a value of 0.8572, which suggests that the inferences and conclusions produced by the present study demonstrate sufficient validity.

Finally, following the data obtained in the process of validation, the resultant questionnaire comprised 25 multiple response items. Results pertaining to the following variables were analyzed: socio-demographics (sex, course, number of siblings, with whom respondents live, nature of the relationship with parents, etc.). We collected data which highlighted the integration of respondents within the educational center (friendships, peer relationships, the teacher-pupil relationship, etc.). The questionnaire continues with questions related to the topic of intimidation (whether respondents had been the victim of intimidation, the individuals who intimidate, capacity for intimidation, forms and frequency of intimidation, role of the bully, role of the teacher). Finally, questions were posed about the conflictive situations in the center and the possible solutions on the part of the educational community.

### 3.3. Procedure

In order to develop the instrument designed for the present study, conceptualization of the aspects to be measured was analyzed by conducting a comprehensive bibliographic review which centered on the extant literature relevant to the topic. The bibliography employed in the development of the present study was selected due to its relevance to the specific theme. This supplied information concerning the current state of the question and enabled the identification of key variables found in situations of school or cyberbullying including, role involvement, types of violent conducts and the school context in which they are produced.

Following this, an interview was conducted with the Management Team of the Secondary and baccalaureate Education Institute to propose the idea of carrying out a research study in relation to the present subject matter and requesting permission to conduct it. After gaining permission from the Management Team, received during both a staff meeting and from the School Board, to conduct the study, teachers of each class were provided with the pupil questionnaires. Questionnaire completion by pupils was voluntary and the days for data collection were selected to coincide with the tutorial hour of each group, in the case of participants in compulsory secondary education, and with an hour of the tutor’s lesson, in the case of baccalaureate participants.

Pupils completed the questionnaires in their classroom. The class tutor and a member of the research team were on hand at all times to explain the study objectives and planned outcomes, and give instructions on correct questionnaire completion. Participants required approximately 20 min to complete the questionnaire, except in the case of pupils with specific educational support needs who had additional time.

## 4. Results

The cross of the variable ‘with whom do you live?’ and the variable describing sex suggests that responses given by males and females were evenly balanced, with significant differences in relation to these dimensions not being found, suggesting that these dimensions could be treated independently.

Analysis of the perceived relationship between pupils and their parents showed that 94.4% of participants in the present study felt this to be good or excellent, with this relationship being very rarely reported as being bad (2.3%) or very bad (0.4%). No correlation existed between this variable and the sex of the pupil, and, thus, these variables are treated independently.

With respect to the number of “good friends” counted on by the respondent at their school, 50% identified at least six friends. On the other hand, in the investigation of cases of isolation at school, participants were asked to report the frequency of occasions on which they had felt alone during break time due to rejection from their friends. A total of 73.5% of pupils indicated that they had never felt alone during break time; however, 2.5% of pupils reported having felt alone many times.

Regarding the perception of treatment received by teachers, it should be noted that 69.3% of students who participated in the present study reported that their teachers treated them well or very well. Only 3.6% of pupils indicated that their teachers treated them badly or very badly. A dependent relationship was uncovered between this variable and gender of pupils. This was demonstrated by a significance value lower than 0.05 being obtained from chi-squared analysis.

General performance in the school was reported by pupils as being good (50%). Nevertheless, attention must be paid to the 23.1% of pupils who considered performance to only be average. Further, this variable is correlated with the variable describing sex, with females generally being more optimistic than males in their perception of their performance at school.

In order to examine school bullying further, pupils were asked their opinions regarding this phenomenon and about its possible causes. A total of 55.2% of participants in the present study expressed that they had never been intimidated by anybody at any time, though the 14% of participants who reported that they “did not know” should also be acknowledged. In relation to individualization of bullies, 18.2% indicated that bullying took place within a group of males, with 2% expressing the opinion that it took place within a group of females. However, in relation to this variable, the majority of respondents indicated that they did not know (28%) or that they did not recognize victimization to have occurred, suggesting that there was nobody who assumed this role at their school (26.2%). A dependent relationship exists between this variable and pupil’s gender, evidenced by the chi-squared significance value which was found to be lower than 0.05.

The way in which respondents act when they are victimized supports the aforementioned findings in that 48.6% of participants reported that nobody intimidated them. It is noteworthy that 20.6% reported having spoken about victimization with their peers and 14.4% had discussed it with their relatives.

Responses regarding the likelihood of individuals ever assuming the role of a bully differed greatly in the present study. While 50% indicated that they would never be bullies, 24.5% conceded that they could become a bully should they be provoked. When relating this variable with the variable describing sex, a dependent relationship was found; specifically, of the 50.4% who indicated that they would never bully a classmate, 31.8% were female and 19.6% were male. Participating in situations of intimidation was also justified by 11.2% who highlighted that they had done so as a result of feeling provoked by their peers. chi-squared analysis demonstrated a significance level below 0.05 which denotes the existence of a relationship between sex and participation in acts of bullying.

With respect to the most frequent form of intimidation, 46.4% expressed that they had name-called or ridiculed their peers. Males and females referenced different behaviors suggesting that the variable of sex influences the type of behavior enacted. In this regard, rejection as an explored mode of intimidation was reported by 15.2% of females, being a behavior enacted by only 6.5% of males. On the part of males, name-calling or inflicting physical damage, were more prevalent behaviors than reported in the female sample. With regards to school bullying, 63.4% of included students indicated that such acts occurred infrequently at school; however, perceptions varied as a function of the gender of the pupil responding with a positive correlation existing between both dimensions.

The response to acts of bullying by those who had witnessed them was varied within the studied population and also followed different trends according to sex. In response to seeing bullying taking place, 15.5% reported not having done anything, of which 11.4% were male. 31.2% indicated that they did not do anything but considered that they should have intervened in some way, of which 18.1% responding in this way were female. 26.5% reported giving a warning, with 15.5% of these being female, while 26.7% revealed that they tried to actively and personally stop the situation. In this way, the existence of two opposing trends that separate those who act and those who do nothing to prevent or avoid situations of bullying is highlighted.

In addition, pupils were asked whether or not they considered bullying to be a problem that needed to be resolved. In response to this, the majority of respondents emphasized that it was necessary to solve this problem (78.3%). When considering the role of teachers in the resolution of cases of maltreatment between peers, 20.2% reported teacher intervention to be non-existent, suggesting that they never intervene. While opinions in reference to this dimension varied greatly, with a further influence of pupil gender, the results, on the whole, indicate that teacher intervention is forthcoming when faced with these types of acts.

In relation to prevalence, it must be highlighted that 21% of students reflected upon having felt persecuted, harassed, or intimidated by other students in a prolonged way on at least one occasion. On the contrary, 18.1% reported that at some point in time they themselves had acted like a bully, persecuting, harassing or intimidating others. Of these, 11.2% were male and the rest (6.9%) were female.

According to 68.6% of respondents, the resolution of bullying episodes falls on the shoulders of both teaching staff and students, who need to cooperate to resolve conflicts that arise in the school. Further to this, 71.8% of respondents reported that both teachers and students resolve conflicts in practice.

The most frequently reported types of aggression were verbal in nature, consisting of insults, threats, or picking on somebody, with this being reported by 46.7% of participants in the present study. Alternatively, 26.5% reported acts of social isolation and rejection to be the most common forms of aggression.

Conflicts were considered to be infrequent problems by 47.7% of respondents, whilst 20.9% reported that they were relatively frequent problems. Resolution of conflict arising from coexistence in schools, was reported by 35.4% of those surveyed to occur through the meting out of punishments or sanctions to penalize conflictive behaviors.

According to 90.8% of pupils, relationships and communication between peers were typically rated as being between average or very good, with very few pupils reporting this aspect to be barely satisfactory (7.2%) or bad (2%). Perception of the school was largely good (35.7%) or normal (45.1%), while 11.7% reported perceiving their school to be horrible or bad.

The present study identified that opinions regarding some aspects of bullying change as young people progress through school and move into different school years. In this regard, analysis of bad words used in lessons revealed different percentages as a function of the school year, with use being very frequent in years one and two of compulsory secondary school education. On the other hand, bad words were reported to be never or rarely used by pupils in their second year of baccalaureate education. Similarly, an evolution in views can be seen with respect to established norms. In the case of pupils undertaking their first and second year of compulsory secondary education, low levels of respect were reported. This contrasts with the findings observed in pupils in their second year of baccalaureate education who reported always being respectful. In the same way, similar observations could be made with regards to the prevalence of insults at the school, fights (although in respect to this dimension, differences according to school year were more subtle), the presence of groups who get along well together, pupils who find themselves isolated and demotivation.

Using bivariate analysis the present study was able to detect that confrontations between pupils and teachers were more habitual in the second year of compulsory secondary education, with 29.6% of pupils suggesting that these clashes occurred often. A total of 25.5% of pupils attending their first and second year of baccalaureate education indicated that teacher–pupil confrontations occurred regularly, while 26.7% reported that they rarely happened. The second most commonly reported opinion of pupils attending second year baccalaureate education was that these confrontations never happened, with this being the view of 26.7% of pupils. Chi-squared analysis of the variables identified them to be independent, suggesting that opinion, in this case, varies as a function of the school year.

The crossing between the variables describing frequency and situation with regards to bad word use in lessons, similarly reflects that the perceptions of pupils in relation to the production of these situations changes as a function of the school year. In this way, 22.4% of pupils attending their first year of compulsory secondary education and 28% of pupils attending their second year of compulsory secondary education indicated that bad words were often used. This was in contrast to findings relating to pupils in baccalaureate education. In this case, 42.5% of students in second year baccalaureate education indicated that bad words had no place in the classroom and 23.8% said they had little place. Equally, chi-squared analysis of these variables reflected that these variables were independent, suggesting that the school year attended by individuals plays a role in transforming opinion.

The same outcomes are produced by crossing the variables describing the frequency and lack of respect for the rules. Specifically, 24.5% and 30.2% of pupils in the first and second year of CSE, respectively, indicated that the rules were often disrespected. The inverse of this was seen in the responses of pupils in the second year of baccalaureate education, who reported that 41.5% never disrespected the rules. The frequency of insults was also examined, with first and second year CSE pupils mentioning that insults had no place at school (20.3% and 30.5%, respectively), whilst 31.8% of second year baccalaureate pupils indicated that insults were never used at school. In relation to the frequency of fights, pupils in first and second year CSE reported that fights were very frequent (20.0% of first year CSE pupils and 34.3% in second year pupils). Perceptions of fight frequency decreased drastically as pupils progressed up the school years, with views appearing to be most different in the first year of baccalaureate education. At this school stage, 29% of pupils reported that fights never took place at school.

In relation to the bivariate analysis of the variables describing groups who get along well together and frequency, it can be observed that 23.8% of pupils in the first year of CSE considered that this situation never occurred. The perception of pupils in second year CSE was identical, with respondents reporting the same score. Alternatively, perceptions in the first year of baccalaureate education were a great deal more positive. In this sense, 31.6% of pupils indicated that the frequency with which groups of students get along well together would be described as often.

The cross of variables describing a lack of integration and frequency produced equivalent outcomes. Perceptions changed as pupils progressed up the school years but trended in the opposite direction, in that 26.2% and 22.6% of first and second year CSE pupils, respectively, reported there to be no lack of integration, whereas first and second year baccalaureate pupils stated that many pupils where not well integrated at school (24.2% of pupils in second year baccalaureate education reported this view).

The commitment of teachers and the frequency with which students felt that teachers “looked after number one” was examined using bivariate analysis. A total of 31.4% of pupils in first year CSE reported that this was never the case. In contrast, 22.3% of pupils in second year baccalaureate education indicated that this was often the case. In relation to the degree of empathy shown by teachers towards their students, 27.5% of pupils in first year CSE believed that their teachers did not understand them at all. This was not the case for first year baccalaureate students, where 22.3% indicated that the teachers understood them a lot. Finally, the cross of the variables describing frequency and student demotivation showed that 31.1% of pupils perceived themselves to not be demotivated at all. This was not the case for pupils in the second year of baccalaureate education, where 23.5% of students indicated that this situation occurred “regularly”.

Results from the exploratory analysis conducted are presented in Table 3 where the situations observed in each school year can be seen. The highest means correspond to the second year of CSE and the lowest means are observed in the second year of baccalaureate education (Table 3).

**Figure 1:**
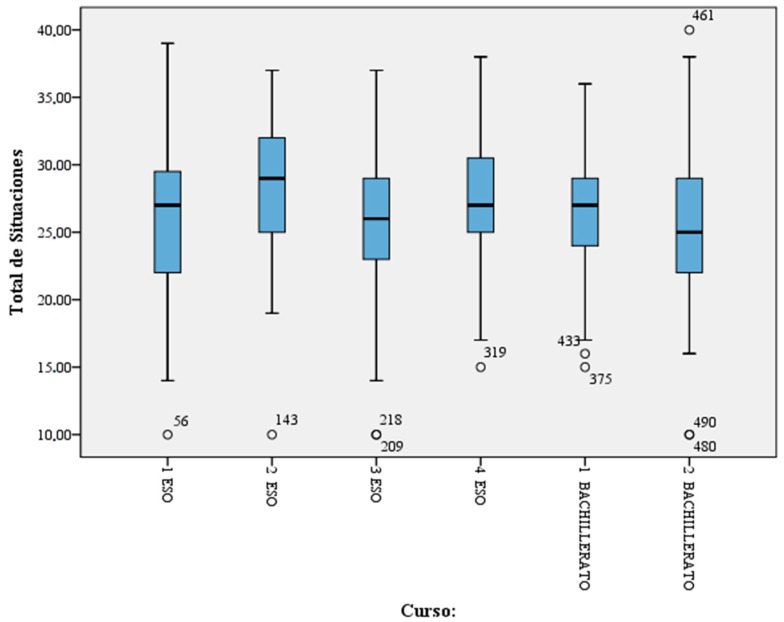
Box plot.

Finally, a box plot graph (Figure 1) is used to visualize the data obtained in the conducted analysis.

It can be seen that the school stage reporting the highest scores are observed in second year CSE. From this the following situations appear to arise:

(1) Confrontations between pupils and their teacher
(2) Use of bad words in class
(3) Disrespecting the rules
(4) Insults coming from students
(5) Fights between students
(6) Students who are not integrated and feel alone
(7) Teachers look after themselves
(8) Students believe that their teachers do not understand them
(9) Pupils are demotivated and bored

It should be noted that some extreme values, also called outliers were found. According to Ross (2007) [30], these are values in statistics described as atypical values in which an observation is numerically distinct from the rest of the data. Statistics derived from datasets that include atypical values need to be frequently revised as they could exert a disproportionate influence on the mean value. This problem is not present in the box plot graph provided as this has been developed using calculations of the median, which is a robust measurement that is less susceptible to the influence of extreme values [30].

## 5. Discussion

School bullying is a problem that is seen in any school context and brings with it serious consequences making it essential to conduct research on the factors that may assist in stopping it [31,32]. In the school investigated, school bullying had a reported prevalence of 21%. This suggests that, through the investigated school, the present study examined a relatively conflictive environment in comparison to the rates of bullying previously reported in Spain, which is quantitatively similar to those found in the USA and other European countries.

The existence of a multitude of methodological approaches in the exploration of this phenomenon could explain differences with regards to the prevalence identified in the various social and geographical environments studied. It is not the purpose of the present paper to examine this deeper; however, as has been discussed by Chaves et al. (2016) [12], it should be emphasized that this is a phenomenon of great magnitude and dynamism that, firstly, poses a challenge to the education system and, secondly, to society in general.

In line with Méndez, Ruiz-Esteban, and López-García (2017) [31] there are three recognized profiles that pertain to school bullying, namely, the aggressor, the victim, and the victim who provokes. Additionally, other studies such as those conducted by Chaves et al. (2016) [12] have highlighted the importance of the spectator as an agent who legitimizes the behaviors of the aggressor or even acts as an accomplice. In the present study, 18.1% of students in the school recognized themselves as having had participated as an aggressor on at least one occasion and 21% as having assumed the role of the victim.

The variables that were associated with victimization related as much with individual factors as they did with pervasive factors found in the school context, such as the climate or the role played by teachers in this environment. Likewise, the role of families and the way in which they intervene when faced with a situation of this nature, also impacts upon its composition [31]. With respect to the students, they expect certain behaviors from their teachers when acts of victimization take place. As is reflected in the results section, 68.6% of pupils expect their teachers to intervene in order to bring such acts to a halt.

In the present study, the quality of relationships established by individuals with their environment was examined and proved to be one of the indicators influencing vulnerability to school bullying. In this sense, studies conducted by Demaray and Malecki (2003) [33] and the study of Schmidt and Bagwell (2007) [34] have demonstrated that acceptance at school is a protective factor when faced with victimization and endorse developing interventions which promote the integration of pupils, alongside their families, into the educational community.

On the other hand, the study conducted by Cava (2011) [35] demonstrated that those individuals who form and maintain relationships with less solid groups and are isolated by their peers have a greater possibility of suffering school bullying. This is in keeping with findings reported by Andrés and Barrios (2009) [36], who have stated that it is necessary to strengthen coexistence in the school environment. Along the same lines, Acosta et al. (2018) [37] have demonstrated that there are four elements that require attention when analyzing the school climate: attachment relationships, assertiveness, empathy, and integration of the individual in the school. Integration of the individual is one of the aspects most highlighted by the academic literature, being examined in studies such as those developed by Cava (2011) and Andrés and Barrios (2009) [35,36]. Further, integration of the pupil in the school is interconnected with the three aforementioned factors (attachment relationships, assertiveness, and empathy) and the climate constructed in the school originates from the inter-relationship between these four elements.

According to Cava (2011) [35], the stronger the connection of the individual with the members of the different environments in which they participate (family, friends, teachers, etc.), the less vulnerable they will be to situations of bullying. In addition, according to Acosta et al. (2018) [37], these individuals will also be more likely to report that they have been victims of school bullying when such situations take place. Wei and Jonson-Reid (2011) [38] also highlighted in their study that having a group of friends to count on acts as a protective factor against school bullying, protecting as much against verbal aggression as it does against physical aggression. This factor, according to Wei and Jonson-Reid (2011) [38] is more efficient when friendship is reciprocated between both members of the relationship (this is to say, when the individuals who form part of the group confirm its existence).

Amongst the key aspects when developing an intervention, Platero (2015) [39] proposes that an approach should be taken using an inclusive perspective which considers impacts upon attitudes of pupils. Another aspect highlighted by Cava (2011) [35] suggests that interventions should improve the interpersonal relationships forged between the pupil and all those who form part of their social circle, especially members of the educational community.

Downes, Nairz-Wirth, and Rusinaitė (2017) [40] have indicated that multi-level policies focusing on the integration of individuals at their school should be incorporated as imperative methods of action. These policies should involve the following: (1) The generation of organizational and curricular strategies targeting the classroom climate, (2) the running of activities to strengthen relationships that incentivize social participation in all groups, (3) teacher training on diversity and improving the classroom climate, and (4) the development of interprofessional networks where teachers, together with other professionals, can pose doubts and difficulties in the pursuit of deepening understanding.

Equally, Vitoroulis, Brittain, and Vaillancourt (2016) [41] demonstrated that the more inclusive an educational center, the lower the rate of victimization, leading to calls to work further on this dimension. In this study, the authors analyzed the influence of ethnicity on the prevalence of school bullying and revealed that this factor was not influential when the policies of the school/center favored integration of their pupils.

According to Smith et al. (2004) [42], in contexts where the educational center or school can provide meeting points to promote interaction between pupils and favor the generation of new and diverse friendships, all of those implicated will become aware of diversity aspects. Downes, Nairz-Wirth, and Rusinaitė (2017) [40] have explained that involving families and converting the school into a social hub will result in a reduced prevalence of bullying as the entire educational community will be more involved and aligned towards achieving the objective of combatting school bullying.

Similarly, Cava (2011) [35] recommend promoting the development of support networks which work towards overcoming situations of victimization suffered in the school environment, offering communication and complaint channels that favor adequate coping.

Smith et al. (2004) [42] have explained that one of the most useful coping mechanisms when encountering bullying is the establishment of a protocol channeled through a system to which victims can resort. Such a strategy can be complemented by the establishment of support networks, as previously defined. In this regard, in the present study, while percentages were found to be low, a group of pupils was identified that reported not receiving adequate treatment from their teachers. This highlights the need to strengthen relationships between all individuals who form a part of the educational community.

Jointly, attention must be given to the tools learned by students to overcome the conflicts that arise in daily life and used to detect if these approaches can be improved using conflict resolution techniques based on relationship improvement [43,44].

## 6. Conclusions

By way of conclusion and following on from the thesis written by Maunder and Crafter (2017) [45], the most effective interventions are those that focus on the school or educational center and involve the entire educational community. They also take a transversal approach leading to a transformation of the cultural system in which the pupils develop. Méndez, Ruiz-Esteban, and López-García (2017) [31] insist that any intervention program developed to overcome school bullying must be designed to connect risk factors with protective factors and examine other risk behaviors, such as alcohol or drug consumption, with the aim of encapsulating the problems experienced in adolescence. The same idea is subscribed to in the present article. H1 was supported in the present study as the analysis suggested that contextual factors gain weight in the production of events that take place in the school. Further, in connection with these findings, interventions should be generated that are developed based on the factors present in the environment in which the intervention will be delivered to prevent school bullying. Vivolo, Holt, and Massetti (2011) [46] also support this outlook and have established that additional individual factors should be considered. It is clear that when interventions are built to be based on contextual factors and the personal conditions of pupils present in the environment, approaches achieve maximum effectiveness.

A limitation of the present study is the difficulty of identifying causal relationships given that only descriptive results can be produced through the methodology employed. Future research could produce more meaningful results by conducting longitudinal studies examining the variables studied here.

## Figures and Tables

**Table 1:** Frequencies for the demographic variables according to sex.

Gender		Frequency	Percentage	Valid Percentage
Valid	Male	268	48.4	48.4
	Female	286	51.6	51.6
	Total	554	100.0	100.0

**Table 2:** Frequencies for the demographic variables according to level of education.

Course		Frequency	Percentage	Valid Percentage
Valid	1 CSE	84	15.2	15.2
	2 CSE	93	16.8	16.8
	3 CSE	78	14.1	14.1
	4 CSE	68	12.3	12.3
	1 BACCALAUREATE	125	22.6	22.6
	2 BACCALAUREATE	106	19.1	19.1
	Total	554	100.0	100.0

CSE: Compulsory Secondary Education.

**Table 3:** Descriptive statistics relating to the present sample.

Course		Mean	Median	Mode	Standard Deviation
School year	1 CSE	23.42	24.00	24.00	5.21
	2 CSE	25.35	26.00	24.00 ^a^	4.64
	3 CSE	22.60	22.00	22.00	4.69
	4 CSE	23.99	24.00	24.00	4.50
	1 BACCALAUREATE	23.05	23.00	23.00	3.96
	2 BACCALAUREATE	22.51	22.00	21.00 ^a^	4.85
	Total	23.44	23.00	23.00	4.70

^a^ Multiple modes exist. The smallest value is shown. The variable Situation has not been included in this total score: “There are groups that get along well together”, as this is a variable that could be considered positive or inverse depending on other situations that highlight the negative.
